# Strategic Lesions in the Anterior Thalamic Radiation and Apathy in Early Alzheimer's Disease

**DOI:** 10.1371/journal.pone.0124998

**Published:** 2015-05-01

**Authors:** Mario Torso, Laura Serra, Giovanni Giulietti, Barbara Spanò, Elisa Tuzzi, Giacomo Koch, Carlo Caltagirone, Mara Cercignani, Marco Bozzali

**Affiliations:** 1 Neuroimaging Laboratory, Santa Lucia Foundation, IRCCS, Rome, Italy; 2 Department of Clinical and Behavioural Neurology, Santa Lucia Foundation, IRCCS, Rome, Italy; 3 Department of Neuroscience, University of Rome ‘Tor Vergata’, Rome, Italy; 4 CISC, Brighton & Sussex Medical School, Brighton, United Kingdom; Nathan Kline Institute and New York University School of Medicine, UNITED STATES

## Abstract

**Background:**

Behavioural disorders and psychological symptoms of Dementia (BPSD) are commonly observed in Alzheimer’s disease (AD), and strongly contribute to increasing patients' disability. Using voxel-lesion-symptom mapping (VLSM), we investigated the impact of white matter lesions (WMLs) on the severity of BPSD in patients with amnestic mild cognitive impairment (a-MCI).

**Methods:**

Thirty-one a-MCI patients (with a conversion rate to AD of 32% at 2 year follow-up) and 26 healthy controls underwent magnetic resonance imaging (MRI) examination at 3T, including T2-weighted and fluid-attenuated-inversion-recovery images, and T1-weighted volumes. In the patient group, BPSD was assessed using the Neuropsychiatric Inventory-12. After quantitative definition of WMLs, their distribution was investigated, without an *a priori* anatomical hypothesis, against patients’ behavioural symptoms. Unbiased regional grey matter volumetrics was also used to assess the contribution of grey matter atrophy to BPSD.

**Results:**

Apathy, irritability, depression/dysphoria, anxiety and agitation were shown to be the most common symptoms in the patient sample. Despite a more widespread anatomical distribution, a-MCI patients did not differ from controls in WML volumes. VLSM revealed a strict association between the presence of lesions in the anterior thalamic radiations (ATRs) and the severity of apathy. Regional grey matter atrophy did not account for any BPSD.

**Conclusions:**

This study indicates that damage to the ATRs is strategic for the occurrence of apathy in patients with a-MCI. Disconnection between the prefrontal cortex and the mediodorsal and anterior thalamic nuclei might represent the pathophysiological substrate for apathy, which is one of the most common psychopathological symptoms observed in dementia.

## Introduction

Amnestic mild cognitive impairment (a-MCI) is defined as a clinical condition characterized by memory deficits in isolation or associated with additional cognitive dysfunctions in the absence of dementia [1]. In a proportion of cases, a-MCI is associated with an increased risk for conversion to dementia in a short time, and is therefore regarded as a “prodromal” stage of Alzheimer’s disease (AD) [2]. In MCI, cognitive deficits as assessed by neuropsychological instruments have, by definition, to be present, thus allowing fulfillment of current diagnostic criteria [3]. In addition, behavioural disorders and psychological symptoms of Dementia (BPSD) are also frequently observed in the AD spectrum [4] with depression, apathy and irritability as the most frequent manifestations [5]. BPSD has been shown to have a strong impact upon patients’ disabilities and quality of life [6], and previous studies have supported the hypothesis that neuropsychiatric symptoms, particularly depression, apathy, and anxiety, are associated with an increased risk for conversion to dementia [7]. In this view, a few functional [8] and structural neuroimaging investigations [9, 10] have attempted to clarify whether there is a neurobiological substrate for BPSD in AD and MCI [10]. At least two of these studies have found a strict association between the presence of specific BPSD and the severity of grey matter (GM) atrophy in brain areas typically affected by AD pathology [9,10]. In detail, GM atrophy involving the fronto-parietal regions was found to be associated with apathy and agitation [9], while atrophy of the anterior cingulate cortex was found associated with disinhibition [10].

On the other hand, white matter (WM) tissue damage is also known to occur in AD pathophysiology and to partially account for patient clinical features [11]. Several previous studies, based upon quantitative magnetic resonance imaging (MRI) techniques, have detected the presence of microscopic WM changes, which in some cases correlated with patients’ measures of cognitive decline [12,13]. In patients with neurodegenerative dementia, the presence and extent of macroscopic WM lesions is usually larger than in healthy elderly individuals [14]. The pathological substrate underlying these macroscopic lesions still remains under debate, and two major hypotheses have been proposed so far. According to the first hypothesis, WM lesions might reflect the occurrence of cerebrovascular comorbidity [15]; whereas according to the second they might be due to cerebral amyloid angiopathy [16]. Regardless of the pathophysiological substrate of WM lesions, it is conceivable that they might contribute to determining some patients’ clinical features when their anatomical localization is within strategic areas. There are several methods to assess the anatomical distribution and severity of WM lesions, and to correlate them with clinical variables. One useful approach, called voxel-lesion-symptom mapping (VSLM) [17] allows, after detection of WM lesions, to associate their anatomical localization with patients’ clinical, neuropsychological and behavioural measures. VSLM has already been successfully used in patients with stroke [18] and brain injury [19].

The aim of this study was to assess, using VSLM, whether specific anatomical localizations of macroscopic WM lesions might contribute to determining BPSD in patients with a-MCI.

## Methods and Materials

### Subjects and experimental procedure

A cohort of 31 patients responding to the diagnostic criteria for amnestic MCI [1] (15 of them with a-MCI single domain and 16 with a-MCI multiple domain) was consecutively recruited from the Specialist Dementia Clinic of Santa Lucia Foundation (Table 1). All MCI patients included here belong to a larger consecutive population participating in a longitudinal, ongoing study, with clinical and neuropsychological follow-up regularly performed at six monthly intervals plus or minus 2 weeks, over 2 years. At the time of recruitment a-MCI patients (both single and multiple domain) had to report subjective memory impairment at clinical onset, corroborated by an assistant and confirmed by performances below the cut-off scores of normality on at least one of the administered tests for episodic memory (see Table 2). Additionally, patients with a-MCI multiple domain had to report pathological scores in at least one cognitive function different from memory. By definition, all MCI patients had not to fulfill the Diagnostic and Statistical Manual of Mental Disorders (DSM-IV) criteria for the diagnosis of dementia [20], their MMSE score [21] had to fall above the cut-off for normality (>23.8), and their Clinical Dementia Rating score (CDR) [22] score had to be equal to 0.5. At each follow-up, all patients received clinical and neuropsychological re-assessments. To increase the confidence of including patients in a pre-dementia stage of AD [3], a clinical conversion to AD or an AD-type progression of cognitive impairment had to be proven during follow-up. In the case of “neuropsychological progression”, a-MCI patients single domain had to develop additional deficits in the areas of visuospatial abilities and/or executive functions. Additionally, none of the recruited patients had to develop remarkable language deficits, parkinsonism, or fluctuations.

**Table 1:** Principal characteristics of studied subjects.

	a-MCI	HS
	N = 31	N = 26
Mean (SD) age [years]	71.3 (8.1)	67.5 (7.0)
F/M ratio	14/17	11/15
Mean (SD) MMSE	26.0 (1.6)*	28.6 (1.4)
Mean (SD) Hachinski score	1.3 (1.6)	0.8 (1.3)
Mean (SD) T2-lesion volume [mL]	3.8 (5.8)	1.6 (2.9)
Mean (SD) T2-lesion fraction	0.3 (0.4)	0.1 (0.2)

Abbreviations: a-MCI = amnestic Mild Cognitive Impairment; HS = healthy subjects; MMSE = Mini Mental State Examination.

*a-MCI *vs*. HS p-value≤ 0.05.

**Table 2:** Neuropsychological assessment.

Cognitive domain	a-MCI	HS
Neuropsychological test		
**Verbal episodic long-term memory**		
**15-Words List:**		
Immediate recall (cut-off ≥ 28.5)	30.3 (6.1)*	45.3 (9.3)
Delayed recall (cut-off ≥ 4.6)	4.4 (2.3)*	9.4 (2.1)
**Short story test:**		
Immediate recall (cut-off ≥3.1)	4.7 (1.4)	5.9 (1.5)
Delayed recall (cut-off ≥ 2.6)	3.7 (2.3)*	5.9 (1.3)
**Visuo-spatial episodic long-term memory**		
**Complex Rey's Figure:**		
Immediate recall (cut-off ≥ 6.4)	11.0 (6.9)	14.5 (7.6)
Delayed recall (cut-off ≥ 6.3)	9.9 (7.6)	14.6 (6.5)
**Verbal short-term memory**		
**Digit span** (cut-off ≥ 3.7)	5.2 (0.9)	5.9 (1.1)
**Visuo-spatial short-term memory**		
**Corsi span** (cut-off ≥ 3.5)	4.4 (0.5)*	5.0 (0.9)
**Executive functions**		
**Phonological Word Fluency** (cut-off ≥17.3)	29.8 (6.0)	35.9 (8.7)
**Mod. Card Sorting** Criteria achieved (cut-off ≥ 4.2)	3.8 (1.9)*	5.7 (0.7)
**Mod. Card Sorting** perseverative errs. (cut-off ≤7.6)	6.3 (4.8)	4.8 (4.8)
**Language**		
**Naming of objects** (cut-off ≥ 22)	28.4 (1.8)	29.1 (1.0)
**Reasoning**		
**Raven's Coloured Progr Matrices** (cut-off ≥ 18.9)	27.7 (5.2)*	32.5 (2.7)
**Constructional praxis**		
**Copy of Complex Rey's Figure** (cut-off ≥ 23.7)	29.6 (6.5)	33.0 (2.2)
**Copy of drawings** (cut-off ≥ 7.1)	9.5 (1.7)*	10.8 (1.2)
**Copy of drawings with landmarks** (cut-off ≥ 61.8)	66.3 (4.3)	69.4 (0.8)

(*) One-way ANOVA p-values Bonferroni’s corrected <0.003 (0.05/16)

Abbreviations: a-MCI = amnestic Mild Cognitive Impairment; HS = healthy subjects; Mod. = modified, errs. = errors

For each administered test appropriate adjustments for gender, age and education were applied according to the Italian normative data. Available cut-off scores of normality (> 95% of the lower tolerance limit of the normal population distribution) are also reported for each test.

Within 3 days of recruitment, patients underwent neuropsychological and behavioural assessments and an MRI scan. An extensive neuropsychological battery was used to define each participant’s cognitive profile (Table 2), as previously described [10]. As detailed below, the assessment of behavioural symptoms was performed, in patients only, by means of the Neuropsychiatric Inventory-12 (NPI-12) [23]. Because the NPI-12 can only be used with patients, a trained psychologist administered a clinical interview to the healthy participants in order to exclude the presence of major behavioural disorders. With respect to the presence and severity of macroscopic WM lesions, there are no definite cut-off criteria available to discriminate between vascular and neurodegenerative dementia [15]. A certain overlap between neurodegeneration and vascular pathology is likely to characterize most patients responding to the diagnostic criteria for MCI [3] and AD [24]. However, to reduce the risk of including patients with vascular MCI as much as possible, principal comorbidities (i.e., diabetes, hypertension, hyperlipidemia, arrhythmias) and risk factors (i.e., alcohol abuse) for developing cerebrovascular pathology were ruled out in all patients. Additionally, in every patient, the Hachinski score [25] had to be less than 4. The Hachinski scale is a clinical tool often used to differentiate between AD and vascular dementia, with a cut-off score ≤ 4 being suggestive for AD-type dementia, and a score ≥ 7 suggestive for Vascular Dementia. Moreover, we considered as additional inclusion criteria a total WM lesion volume not exceeding 25 mL [15]. As explained below, 26 healthy elderly volunteers (healthy subjects: HS) were also recruited as a control group for neuropsychological and neuroimaging data (i.e., lesion load and distribution, GM volumetrics). In all HS, the presence of major systemic and neurological illnesses, cognitive deficits (assessed using the same neuropsychological battery used in the a-MCI population), and behavioural disorders (assessed by clinical interviews) were carefully investigated and excluded. Finally, to reduce any potential source of variability due to hemispheric dominance, all subjects had to be right-handed (as assessed by the Edinburgh Handedness Inventory) [26]. Principal demographic and clinical characteristics of all recruited subjects are summarized in Table 1.

### Ethics statement

The Ethics Committee of Santa Lucia Foundation approved the study. Written informed consent was obtained from all recruited subjects before study initiation.

### Behavioural assessment

The Neuropsychiatric Inventory-12 (NPI-12) [23] was used to quantify the presence and severity of patients’ BPSD [7]. Briefly, this scale is a semi-structured inventory completed by the patients’ caregivers and designed to detect the following symptoms: delusions and hallucinations, agitation/aggression, dysphoria/depression, anxiety, euphoria/elation, apathy, disinhibition, irritability/lability, aberrant motor behaviour, sleep and eating disturbances. Each item’s score ranges from 0 to 12, and reflects both severity and frequency of each behavioural symptom, with 0 corresponding to the absence of behavioural symptom and 12 corresponding to its maximum frequency and severity. The frequency rating multiplied by the severity rating produces a score for each behavioural symptom. Consistent with Cummings [27], we applied (subject by subject) the following cut-offs to consider BPSD as clinically relevant: for delusions, hallucinations, agitation, euphoria, apathy, aberrant motor behavior a score ≥1; for irritability a score ≥ 2; for disinhibition a score ≥ 4; for depression a score ≥ 6; Finally, although an NPI cut-off score for clinically relevant anxiety has not been proposed by Cummings, we used a score ≥ 4, as reported in a recent study [28].

Collinearity (i.e. patterns of co-occurrence or correlation) within BPSD was also assessed across patients. In order to evaluate the interdependence between clinically relevant BPSD, we reduced each NPI-12 subscale from continuous to categorical data (presence/absence of symptoms) in accordance with the cut-off reported above. Therefore, each NPI-12 subscale was correlated with each other by using r_phi_ coefficient. To control for the I-type error we used the Bonferroni Correction for multiple comparisons (statistical threshold = p<0.004 [α/n = 0.05/12]).

Spearman’s correlation coefficient was used to assess associations between clinically relevant BPSD and neuropsychological scores (p value set at <0.003 after Bonferroni’s correction [α/n = 0.05/16]).

### Magnetic resonance imaging

All subjects underwent MRI scanning at 3T (Magnetom Allegra, Siemens, Erlangen, Germany), including the following acquisitions: (1) Dual-echo turbo spin echo (TSE) (repetition time [TR] = 6.190 ms, echo time [TE] = 12/109 ms, matrix = 256x192; FOV = 230x172.5mm^2^;); (2) fast-fluid-attenuated inversion recovery (FLAIR) (TR = 8.170 ms, TE = 96 ms, inversion time [TI] = 2.100 ms, same matrix and FOV as the TSE); (3) 3D Modified-Driven-Equilibrium-Fourier-Transform (MDEFT) scan (TR = 1338 ms, TE = 2.4 ms, Matrix = 256x224x176, in–plane FOV = 250x250 mm^2^, slice thickness = 1 mm).

### Assessment of macroscopic white matter lesions

T2-hyperintense lesions were first identified by consensus by two trained observers (M.T. and B.S.) on TSE images. Then, lesions were outlined on the shortest echo TSE sequence using a semi-automated local thresholding contouring technique (Jim 4.0. Xinapse System. Leicester. UK. http://www.xinapse.com/). For each subject, global WM lesion volume and fraction were calculated. WM lesion fraction is defined as global WM lesion volume divided by intra-cranial volume (ICV). For each subject, ICV was derived by adding the volumes of each tissue segment (WM, GM and CSF) obtained by voxel-based morphometry (VBM). WM lesion volumes and fractions were both used to investigate potential associations with those neuropsychological scores for which patients reported significantly different scores than HS, and patients’ BPSD scores. Finally, WM lesion masks were used for VSLM analyses.

### Voxel-lesion-symptom mapping

To investigate the potential association between WM lesion distribution and neuropsychological performance and occurrence/severity of BPSD in patients with a-MCI, we employed the VLSM approach [17]. VLSM is a voxel-wise statistical method that allows a correlation between continuous clinical measures and lesion distribution on a voxel-by-voxel basis. In brief, given a clinical variable of interest (e.g., anxiety NPI subscore), VLSM performs a series of T-tests, one for each voxel included in patients’ lesions, based upon the presence or absence of lesional tissue in each given voxel. This therefore allows the identification of those brain areas whose damage (i.e., presence of macroscopic lesions) has the greatest impact on a certain clinical variable (e.g., anxiety NPI subscore). To perform the VLSM analysis we firstly normalized the lesion mask of every subject to Montreal Neurological Institute (MNI) space as follows. The shortest echo TSE images (also used for lesion outline) was segmented into GM, WM and CSF using SPM8-NewSegment [29]. We then added the three tissue probability maps to obtain a probabilistic brain mask matching the TSE scan. This probabilistic brain mask was thresholded and binarised, and used to skull-strip the original TSE image. The skull-stripped TSE image was then affine transformed (using the tool FLIRT from FSL library, www.fmrib.ox.ac.uk/fsl/) to match the template “MNI152_T1_1mm_brain” (a T1-weighted atlas in MNI coordinates [30] available with FSL). The same transformation was then applied to the binary lesion mask to finally get the lesion mask normalized to the MNI space. All NPI-12 scores with a prevalence of at least 20% in our patient sample were entered as variables of interest in VLSM analyses [17]. For each neuropsychological and behavioural variable taken into consideration, the T-tests were confined to those voxels where at least 4 subjects had lesions in order to maintain a reasonable level of statistical power. The resulting statistical maps were corrected for multiple comparisons at cluster level using permutation tests [31] with 1000 repetitions, accepting as significant p values of less than 0.05.

### Brain Volumetrics

MDEFT scans were used for voxel-based morphometry (VBM) analysis according to the optimized protocol implemented in SPM8 (http://www.fil.ion.ucl.ac.uk/spm/) [29]. Briefly, VBM consists of an iterative combination of segmentations and normalizations to produce a GM probability map [29,32] in MNI space for every subject. In order to compensate for compression or expansion which might occur during warping of images to match the template, GM maps were “modulated” by multiplying the intensity of each voxel in the final images by the Jacobian determinant of the transformation, corresponding to its relative volume before and after warping [33]. GM, WM and CSF volumes were computed from these probabilistic images for every subject. All data were then smoothed using a 12-mm FWHM Gaussian kernel. Statistical analyses were finally performed on smoothed GM maps within the framework of the general linear model. An ANOVA full factorial design was first employed to compare regional GM volumes between a-MCI patients and HS. A multiple regression was used to investigate the potential association between global WM lesion and regional GM volume in patients only. Further, two additional statistical models were respectively employed to assess associations between: 1) regional GM volumes and scores at neuropsychological tests for which a-MCI patients performed significantly worse than HS; 2) regional GM volumes and NPI-12 scores for those BPSD found to be present in our patient cohort.

Finally, additional post-hoc ANOVA models were planned to test for potential regional GM differences between sub-groups of patients with a WM lesion distribution associated to BPSD according to VSLM.

In all VBM analyses, ICV, age and gender were entered as covariates of no interest. Statistical threshold was always set to p-values family-wise-error (FWE) corrected at cluster-level inferior to 0.05.

## Results

According to the inclusion criteria, there were no significant differences in age (F_1, 55_ = 3.47, p = 0.067), gender distribution (Chi-square = 0.05, degree of freedom (dof) = 1; p = 0.82) and Hachinski score (F_1, 55_ = 1.5, p = 0.022) between a-MCI patients and HS (Table 1). As expected, a-MCI patients showed significantly lower MMSE scores than HS (F_1, 55_ = 34.2, p<10^–4^). At clinical follow-up, 10 of 31 patients (32%) converted to AD (according to the current diagnostic criteria [34]), while 21 (68%) revealed the typical neuropsychological progression of AD as defined in the methods. None of them reverted back to normality or converted to other forms of dementia.

### Neuropsychological and behavioral assessment

From a neuropsychological viewpoint, a-MCI patients (both single or multiple domain) revealed impairment of episodic memory and executive functions (Table 2). According to the criteria outlined in the Methods, the following scores were retained for correlating with GM volume, WM lesion volume and VSLM analyses: Immediate and delayed recall of a 15 word list; Delayed Recall of Short Story test; Raven’s Matrices; Corsi Span; Modified Card Sorting Test and Copy of Drawings. In order to achieve a global significance level of 0.05, the threshold for each test was set at p < 0.003, according to Bonferroni correction (0.05/16).

No significant associations were detected within BPSD. In addition no significant correlation was found between BPSD and neuropsychological scores.

**Fig 1:**
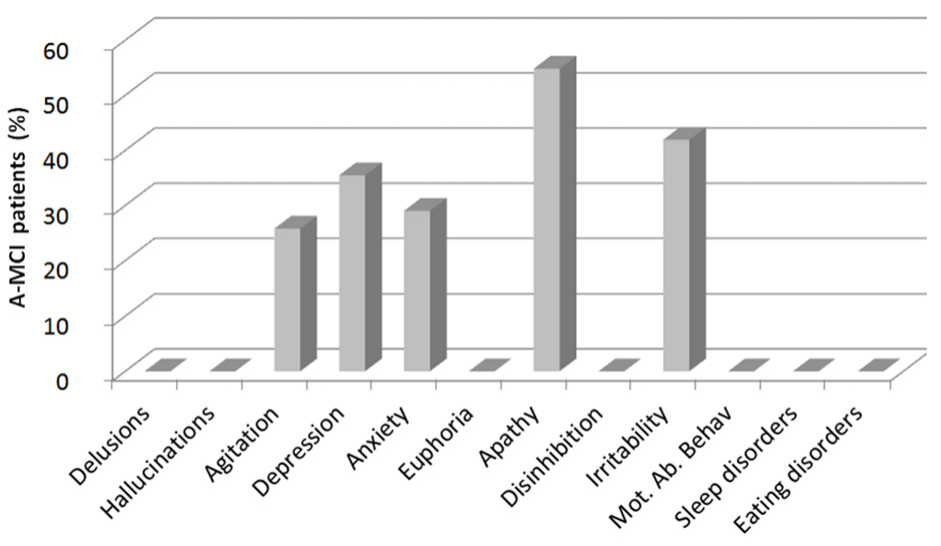
Behavioural disorders and psychological symptoms in amnestic mild cognitive impairment patients. Histograms represent the percentage of amnestic mild cognitive impairment (a-MCI) patients exhibiting each symptom assessed by the Neuropsychiatric Inventory 12 (NPI-12). Abbreviation: Mot. Ab. Behav. = motor aberrant behavior.

With respect to the clinical relevance of BPSD, 17 of 31 a-MCI patients (54.8%) were affected by apathy, 13 of 31 (41.9%) by irritability, 11 of 31 (35.5%) by depression, 9 of 31 (29.0%) by anxiety, and 8 of 31 (25.8%) by agitation (Fig 1). As these 5 BPSD scores were used for correlations with GM and WM lesion volumes and VLSM analyses, the threshold for each test’s statistical significance was set at p < 0.01, according to Bonferroni correction.

### WM lesions

**Fig 2:**
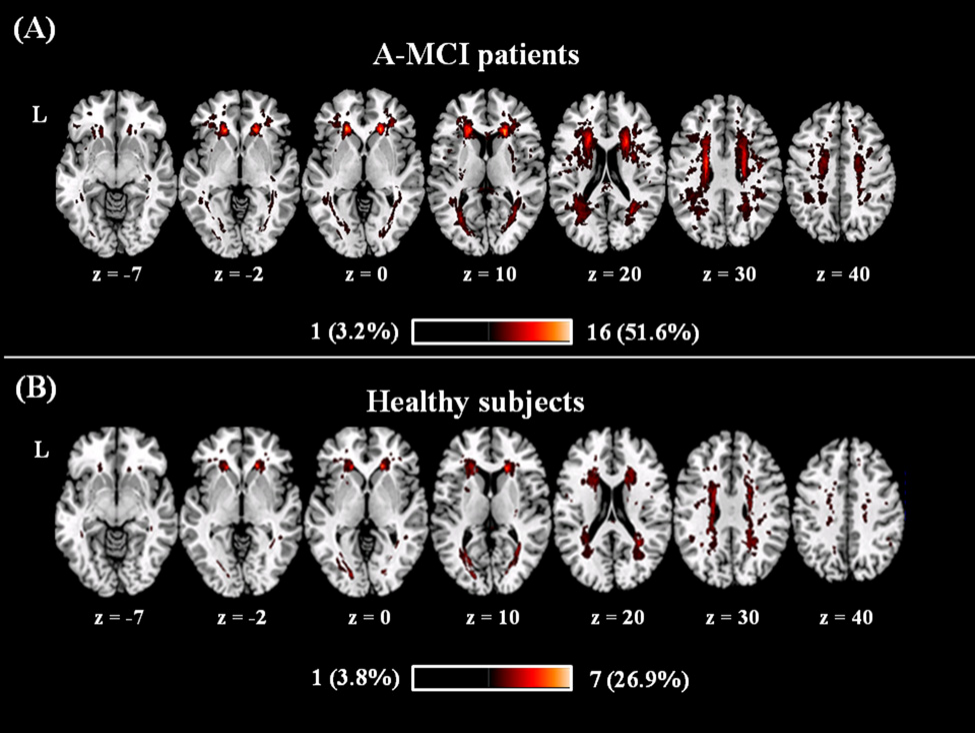
Lesions frequency distribution map in patients (A) and controls (B). Colours represent number of subjects with a lesion to a specific voxel. Warmer areas indicate areas of greater lesion overlap. The anatomical distribution of T2-hyperintense white matter lesions was similar between amnestic mild cognitive impairment (a-MCI) patients (panel A) and controls (panel B). The between-group difference in T2-lesion volumes was not statistically significant, although it was on average slightly larger in the patient group (see also Table 1).

Although the average lesion volume and fraction were higher in a-MCI patients compared to HS (Table 1), this difference was not statistically significant (F_1, 55 =_ 2.8, p = 0.099; and F_1, 55 =_ 3.9, p = 0.053 respectively). The anatomical distribution of WM lesions was similar in the two groups in periventricular areas (Fig 2), although with a greater extension in a-MCI patients.

No significant association was found between patients’ WM lesion volume or fraction, and neuropsychological and BPSD data.

### VLSM analysis

None of the neuropsychological scores for which patients performed significantly worse than HS was found to be associated with WM lesion distribution by VSLM analyses.

**Fig 3:**
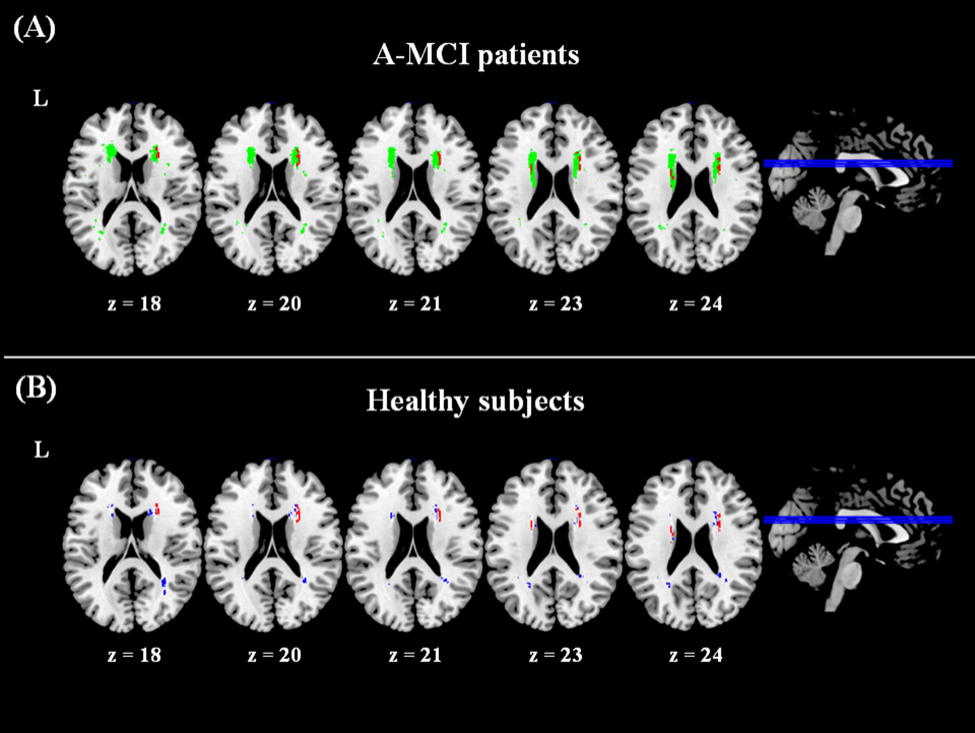
Results of voxel lesion-symptom mapping analyses. A significant association was found between the anatomical localization of white matter lesion in the anterior thalamic radiation (areas in red) and the presence/severity of apathy in between amnestic mild cognitive impairment (a-MCI) patients (panel A). Voxels in green show the lesion distribution in patients used for the voxel lesion-symptom mapping (VSLM) analysis, i.e., only including areas where at least 4 subjects presented a lesion. The lesion distribution in healthy controls (panel B, in blue), does not overlap with the area found to be significant for apathy in patients. Note that the lesion distribution is thresholded to show only voxels where at least 4 participants had a lesion, in order to match those included in the VLSM analysis.

Conversely, a significant association with WM lesion localization in the anterior thalamic radiation (ATR) bilaterally was only found for the NPI-12 apathy score (Table 3 and Fig 3, panel A) only. By using VLSM no other significant associations were found between WM lesions and the remaining NPI-12 subscales.

**Table 3:** Relationship between apathy and WMLs in the a-MCI group as assessed by VLSM.

White matter region	Side	Cluster size	MNI coordinates of max			T-score
			x	y	z	
Anterior thalamic radiation	R	341	24	17	16	4.67
Anterior thalamic radiation	L	80	-22	5	23	5.04

Abbreviations: R = right; L = left; max = relative maximum.

The table shows the areas of white matter lesions found to be correlated with the apathy score by voxel lesion-symptom mapping (VLSM). For each cluster, peak statistics and coordinates are reported. The between groups T-contrast was thresholded with p values<0.05, after correcting for multiple comparisons at cluster level using permutation test. Each region size is expressed as number of voxels. The white matter regions were assigned using JHU-White Matter Tractography Atlas, available in FSL [35]. T-score refers to the value at relative maximum.

The significant WM tracts were labelled as belonging to ATR by using the JHU-White Matter Tractography Atlas [35], available in FSL (http://fsl.fmrib.ox.ac.uk/fsl/fslwiki/). No other significant associations were found. The voxels found to be significantly associated with apathy in patients were also compared with the lesion distribution in HS (Fig 3, panel B), showing no overlap. The two patient subgroups (with and without apathy) were then compared to each other and against HS in all demographic, clinical, and neuroimaging variables. No significant difference was observed between any group (apathetic a-MCI, non-apathetic a-MCI, and HS) for age (F_2,54_ = 1.74, p = 0.19), gender (apathetic a-MCI *vs*. HS: Chi-square = 0.31, dof = 1, p = 0.58; non-apathetic a-MCI *vs*. HS: Chi-square = 0.07, dof = 1, p = 0.79; apathetic *vs*. non- apathetic a-MCI: Chi-square = 0.05, dof = 1, p = 0.82) or Hachinski score (F_1,28_ = 4.04, p = 0.054). The neuropsychological assessment did not reveal any significant difference between apathetic and non-apathetic a-MCI patients. In the clinical follow-up the conversion rate to AD was similar in apathetic (6/17) compared non apathetic (4/14) a-MCI patients (Chi-square = 0.16, dof = 1, p = 0.69). WM lesion volume and fraction were also similar among all groups.

### GM volumetrics

**Fig 4:**
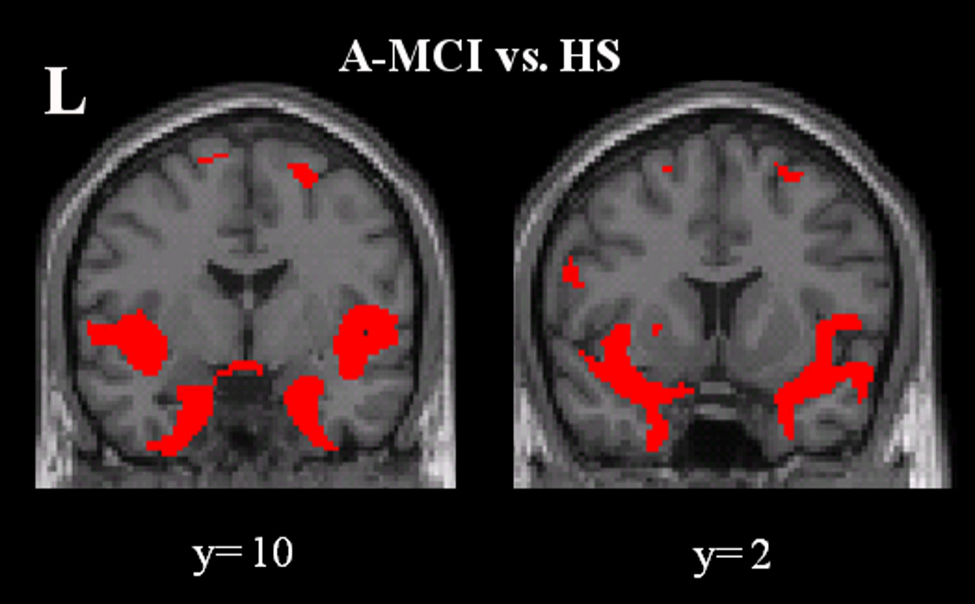
Pattern of regional grey matter atrophy in all a-MCI patients compared to controls. There is shown here the pattern of regional grey matter loss in all a-MCI patients compared to controls. Consistent to previous literature, atrophy was predominantly distributed to the medial temporal lobes. Statistical threshold: p values family wise error corrected at cluster level < 0.05.

When comparing all a-MCI patients to controls, the typical pattern of GM loss dominated by medial temporal lobe atrophy was found (Fig 4).

There was no significant association found between global WM lesion volume and regional GM volumes in patients. Moreover, no significant association was found between regional GM volumes and either neuropsychological or NPI-12 scores in a-MCI patients. Finally, a direct comparison between a-MCI patients with and without apathetic symptoms (the most frequent BPSD in our patient sample) did not reveal any significant difference in regional GM volumes.

## Discussion

In this work, we recruited a consecutive group of patients with a clinical diagnosis of a-MCI due to AD [3]. According to the inclusion criteria, a third of them converted to AD over two years, whilst the remaining ones had a typical neuropsychological evolution for AD. Additionally, patients’ regional pattern of GM loss was consistent with that expected for a-MCI patients according to previous literature [36]. It is well established that macroscopic WM lesions increase progressively with aging [37] and are more likely to occur in the presence of dementia [38]. The pathophysiological substrate of these lesions is still largely controversial. Several previous studies have pointed out that vascular pathologies (e.g.: atherosclerosis, cerebral amyloid angiopathy) and Wallerian degeneration secondary to distal cortical atrophy can often coexist [39–40]. Thus, it is likely that both neurodegenerative process and vascular insults can contribute to injury of cerebral WM. Due to these uncertainties, setting a clear cut-off purely based upon WM lesions volume to distinguish between vascular and neurodegenerative forms of dementia would be too simplistic. In order to minimise the risk of including vascular MCI patients, we ensured their Hachinski ischemic score was extremely low and not significantly different on average from that of healthy controls. WM lesion loads and distribution were also similar between patients and controls [38]. While we did not attempt to clarify the pathological substrate, we hypothesised that WM lesions may contribute to determining brain dysfunctions, especially when localised within strategic areas. In the current work we demonstrated that the strategic localization of WM lesions in the anterior thalamic radiations is highly associated with the occurrence and severity of apathy in combination with AD neurodegeneration, but not of other BPSD or neuropsychological deficits. In support of this specific effect of WM lesion distribution, the comparison of apathetic vs. non- apathetic a-MCI patients did not reveal any other clinical, neuropsychological or brain volumetric difference.

Apathy is a complex syndrome [41] due to direct [42] or indirect involvement of the prefrontal lobes [43], which can be due to either GM or WM damage. In AD patients, atrophy in the anterior cingulate and prefrontal cortex [4,9] as well as WM lesions in the same areas [44] have been found to be associated with apathy. Crucially, the cingulate cortex is strongly connected to the mediodorsal nucleus of the thalamus [45], and disconnection within this circuit has been shown to produce apathetic symptoms [46]. Consistent with this, our results indicate that WM lesions induce apathy by disruption of fibres connecting the thalamus with prefrontal regions. Prefrontal regions are part of a well-known cortico-subcortical network, which is involved in the control of complex human behaviours [5]. Additionally, the anterior cingulate gyrus is a key structure for the regulation of intentional and motivated behaviours [47]. We identified here the bilateral damage of ATR as a strategic location for apathetic manifestations. The ATR consists of fibres connecting mediodorsal and anterior thalamic nuclei to the prefrontal cortex and the anterior cingulate cortex [35].

We did not find any significant association between BPSD (i.e. apathy) and neuropsychological scores. This was unexpected because associations between apathy and executive functions have been previously reported [48,49]. Our failure to detect a relationship in the present study might possibly be explained by the fact that we used the NPI-12 to assess apathy. NPI-12 is a comprehensive tool to assess BPSD that does not allow for the investigation of distinct aspects of apathy (cognitive, motor and affective) [41]. Conversely, it is likely that the association between executive functions and apathy is driven by the cognitive component of apathy [50], which we were not able to isolate in the present study.

Interestingly, we did not find any association between BPSD and GM volume. In a previous study, including both MCI and AD patients [10], we demonstrated a strict relationship between GM volume and positive BPSD, but no association with any negative symptom (including apathy). This is somewhat unexpected, as atrophy of the pre-frontal areas has been previously been implicated in apathy [51]. One of the possible explanations is that the patients included in this study were all at a prodromal stage of AD. We therefore speculate that disconnection within the thalamo-cortical circuits (to which WM lesions contribute) might be initially responsible for apathy, whilst GM atrophy of the frontal lobes usually occurs at a later stage, when more advanced neuropathological processes are likely to take place.

This is a preliminary and exploratory study and suffers from some limitations. First, we only studied the relationship between lesion location and BPSD, and did not attempt to investigate the pathological substrate of those lesions. WM hyperintensities can reflect axonal damage, demyelination, lacunar infarcts, and other forms of vascular damage [15]. It is possible that this these variable causes may result in different clinical manifestations. Consistently, we observed a similar lesion distribution in patients and in HS who did not show any BPSD. Although we did not find any overlap between the lesioned area significant for apathy in patients and the lesion distribution in HS, further investigations, based upon quantitative MRI techniques such as diffusion tensor imaging and magnetization transfer, are warranted, in order to clarify this issue. A further limitation of this study on BPSD is the lack of differentiation of apathy symptoms in sub-components, such as cognitive, motor and affective aspects [41]. Finally, the sample size of this study was relatively small for a VLSM analysis [31]. However, the main consequence is to have a higher chance of false negative findings (type II error), thus supporting the soundness of our results. In conclusion, this study confirms the relevance of WM lesions for some clinical manifestations of AD, since the early phases. This data contributes to supporting the importance of disconnection syndrome, which has been implicated in AD, and typically to explain some cognitive symptoms [52–55]. Here we suggest that disconnection might also be relevant for some behavioural symptoms, namely apathy.
